# Supplementary material for: DMRforPairs: identifying Differentially Methylated Regions between unique samples using array based methylation profiles
Source: BMC Bioinformatics. 2014 May 15;15:141. doi: 10.1186/1471-2105-15-141 (PMC4046028; doi:10.1186/1471-2105-15-141)
Supplement: Additional file 2 — DMRforPairs output for the comparison of A431-MCF7 and NA17018-NA17105. Please start from the HTML files in each folder. Available via the BMC Bioinformatics website. [file 1471-2105-15-141-S2.zip › 1394847754114233_MOESM2_ESM/A431_MCF7/figures/25490.pdf]

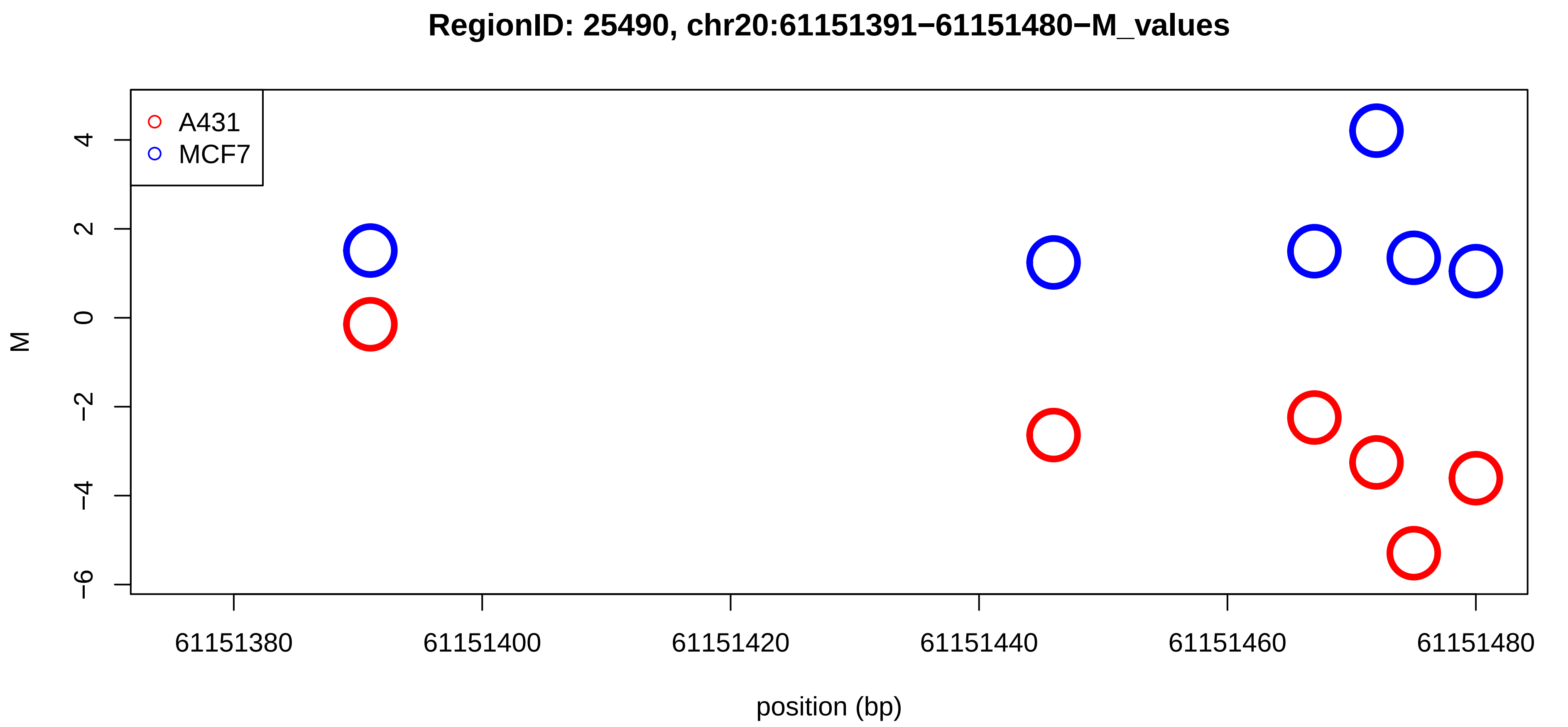
RegionID: 25490, chr20:61151391−61151480−M_values
A431
MCF7
M
4
2
0
−2
−4
−6
61151380
61151400
61151420
61151440
61151460
61151480
position (bp)

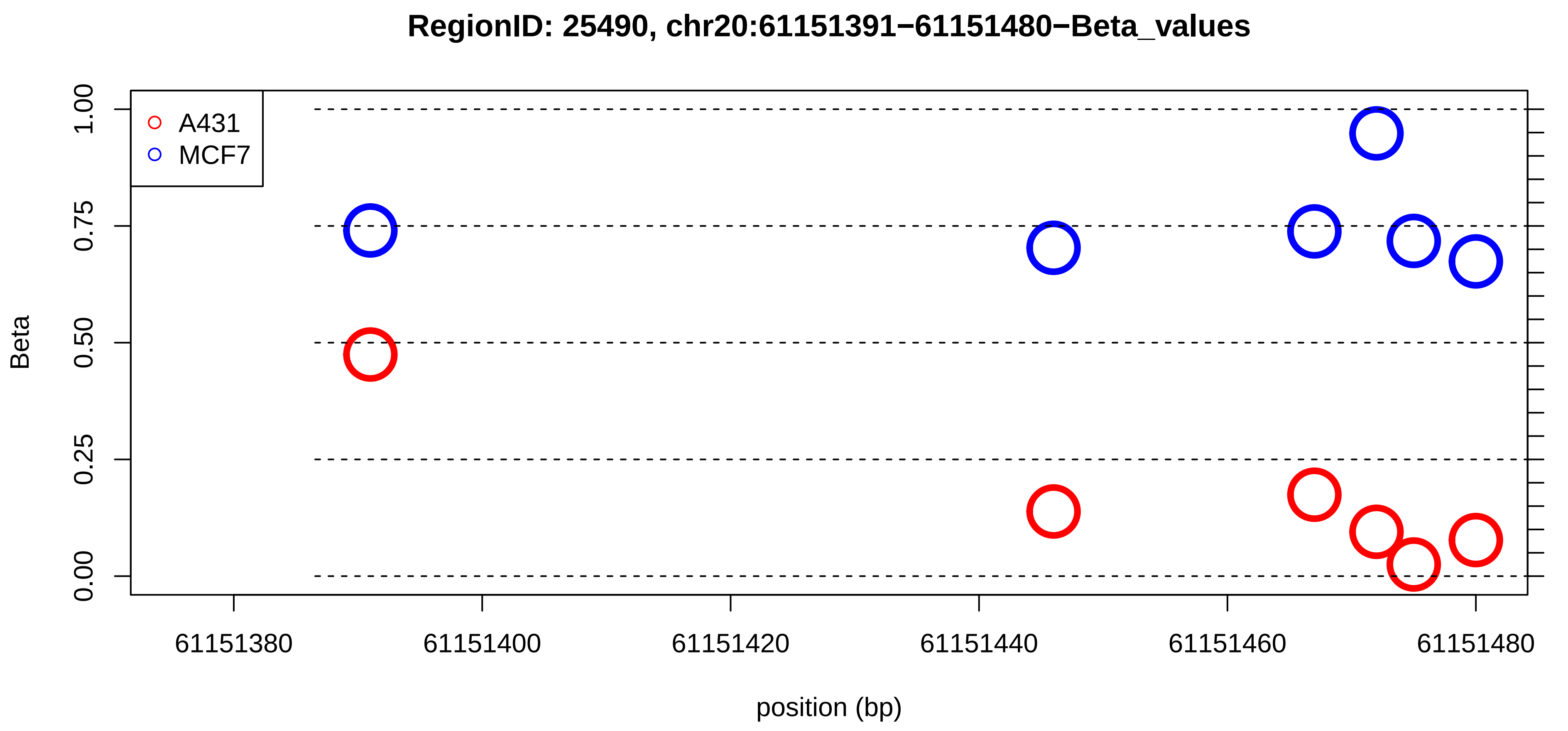
RegionID: 25490, chr20:61151391−61151480−Beta_values
A431
MCF7
Beta
1.00
0.75
0.50
0.25
0.00
61151380
61151400
61151420
61151440
61151460
61151480
position (bp)

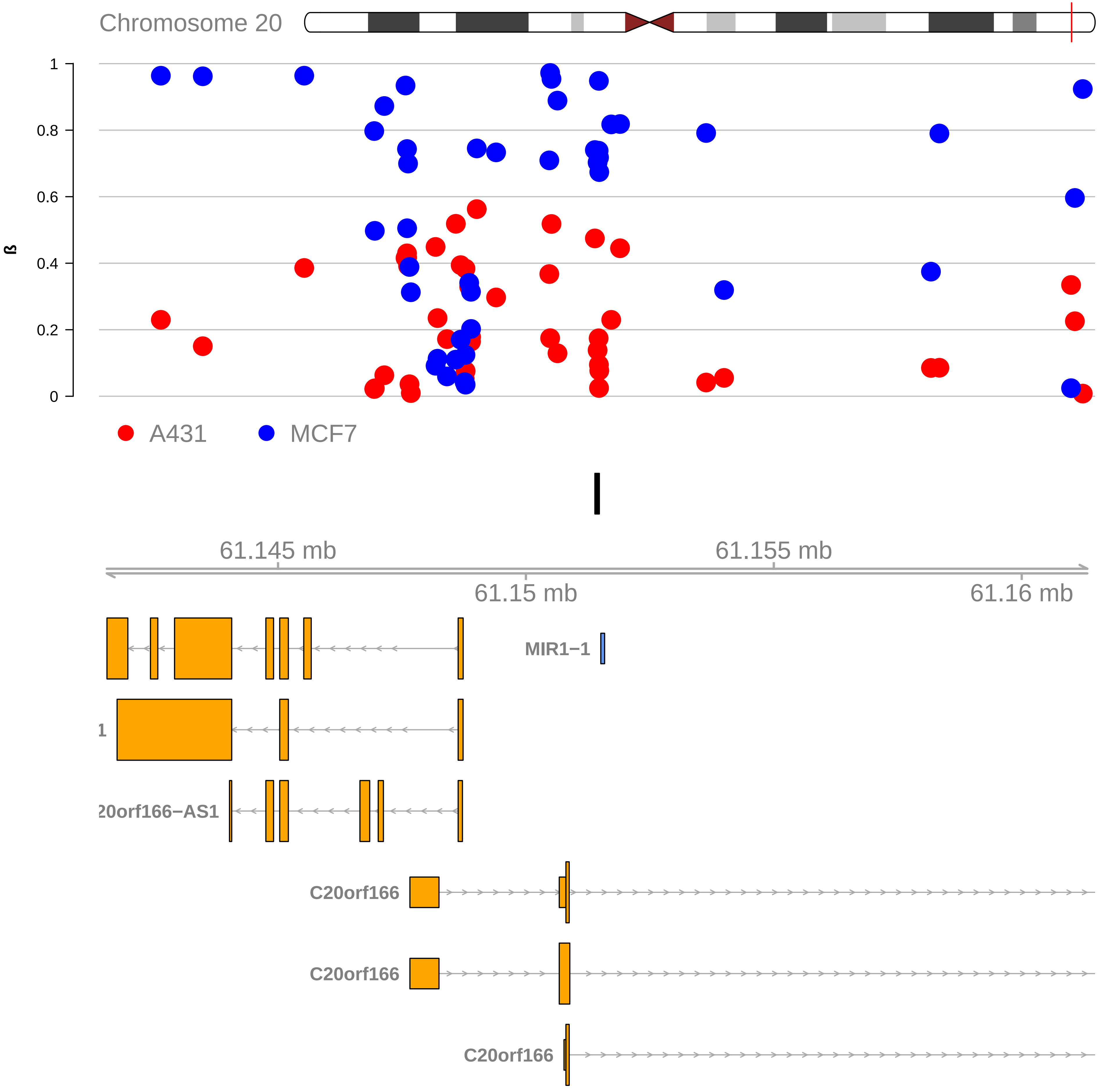
Chromosome 20
β
1
0.8
0.6
0.4
0.2
0
A431
MCF7
61.145 mb
61.155 mb
61.15 mb
61.16 mb
MIR1−1
1
20orf166−AS1
C20orf166
C20orf166
C20orf166
